# Papillary thyroid cancer recurrence 43 Years following Total Thyroidectomy and radioactive iodine ablation: a case report

**DOI:** 10.1186/s13044-017-0043-4

**Published:** 2017-10-10

**Authors:** Yaw Amoako-Tuffour, M. Elise Graham, Martin Bullock, Matthew H. Rigby, Jonathan Trites, S. Mark Taylor, Robert D. Hart

**Affiliations:** 1 Department of Diagnostic Radiology, Dalhousie University, 3rd Floor Victoria Building, VG Site, QEII Health Sciences Centre, 1276 South Park Street, PO BOX 9000, Halifax, NS B3H 2Y9 Canada; 2 Division of Otolaryngology – Head and Neck Surgery, Dalhousie University, 3rd Floor Dickson Building, VG Site, QEII Health Sciences Centre, 5820 University Ave, Halifax, NS B3H 2Y9 Canada; 3 Department of Anatomical Pathology, Dalhousie University, 1459 Oxford Street, Halifax, NS B3H 4R2 Canada

**Keywords:** Papillary thyroid cancer, Thyroidectomy, Radioiodine ablation, Recurrence, Neck dissection, Case report

## Abstract

**Background:**

Recurrent papillary thyroid carcinoma (PTC) beyond the first two decades of definitive treatment (i.e. total thyroidectomy and radioactive iodine ablation) is a rare occurrence.

**Case presentation:**

We present a case of a 71-year old Caucasian female with a distant history of PTC treated with total thyroidectomy and radioactive iodine ablation who experienced recurrence of her disease 43 years following initial diagnosis and definitive treatment. She presented with palpable left-sided neck mass and subsequently underwent a level II, III, neck dissection and adjuvant iodine ablation. This case presents the latest recurrence in papillary thyroid cancer documented to date in the literature.

**Conclusion:**

This case exemplifies the need for the head and neck surgeon, radiation oncologist, general practitioner and radiologist to consider new lateral neck mass as late-presenting recurrence of PTC until proven otherwise regardless of low recurrence rates beyond two decades from treatment and low prognostic risk scores.

## Background

Papillary thyroid carcinoma (PTC) is the most common histological type of thyroid-originating malignancy comprising 80% of all thyroid carcinomas [1, 2]. PTC is an unencapsulated tumor with papillary and follicular structures characterized histologically by overlapping cell nuclei with ground glass appearance and invaginations of cytoplasm into the nuclei [3]. It differs from the follicular variant in the absence of nuclear changes in the latter. These tumors are indolent and have a good prognosis, but frequently metastasize to regional lymph nodes in 5.4% to 13% of patients after initial surgery [1]. As a whole, thyroid cancer generally has a good prognosis with a 5-year survival rate of 98% [4, 5]. The cornerstone of treatment for PTC patients is total thyroidectomy with post-operative radioactive iodine adjuvant therapy [4, 6]. Recurrence of PTC may be loco-regional or distant, with the central compartment typically being the first region to develop metastatic disease, followed by ipsilateral lateral neck nodes [7].

In a study spanning 6 decades (1940-1999) of PTC treated at the Mayo Clinic, recurrence rates followed a logarithmic trend, meaning that the incremental risk of recurrence decreased in each subsequent year [2]. The tumour recurrence rates were 8% at 5 years and 11% at 10 years for the subgroup of 2305 who had localized disease that was completely excised at the initial surgery. By 25 years, the cumulative recurrence rate reached 11% and increased to 13% at 40 post-operative years [2]. More than 80% of recurrences took place during the first decade.

Though most recurrences occur within the first 10 post-operative years, there have been reports of loco-regional recurrences a decade or more after this initial window. Cirocchi reports that local relapses can occur as late as 20 years after the initial diagnosis and treatment [7, 8]. In another report by Schlumberger et al., the authors report a recurrence of differentiated thyroid carcinoma 33 years after initial treatment [9]. In a study of 269 patients which was designed to determine the length of follow-up needed for post-op thyroid patients, the authors found that the latest recurrences happened between 30 and 39 years [10]. The current report presents the case of a loco-regional (lateral neck) recurrence of PTC 43 years after initial treatment. To the authors’ knowledge, this represents the latest PTC recurrence reported in the literature.

## Case presentation

A 71 year old Caucasian female with a remote history of PTC presented with a 2-month history of palpable left-sided neck mass. Physical examination revealed a non-tender mass in the left lateral neck. There were no other abnormalities identified in a full head and neck examination. There was no palpable mass in the thyroid bed. Ultrasonography of the neck revealed an absence of normal thyroid tissue, consistent with patient’s history of distant thyroidectomy, and a solid mass in the left side of the neck superolateral to the expected location of the thyroid, in level III. This inhomogeneous mass appeared vascular and measured 2.8 cm in length by 1.7 cm in diameter. It was not felt to resemble a normal lymph node in ultrasonographic features. Nonetheless, from its appearance and anatomical location, this new mass was initially suspected to represent a lymph node or a carotid body tumour.

**Fig. 1:**
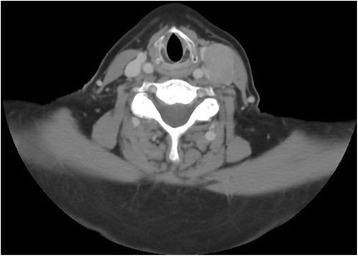
Computed tomography (transverse plane) revealing 2.8 × 1.7 cm mass in patient’s left lateral neck

**Fig. 2:**
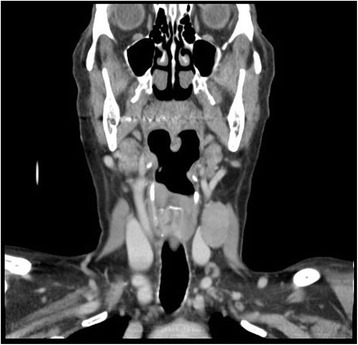
Computed tomography (coronal plane) of the patient’s left lateral neck mass

Computed Tomography (CT) ruled out non-nodal neck pathology, and identified the mass as being consistent with an enlarged lymph node (Figs. 1, 2). Again, this study revealed no evidence of recurrence in the thyroid bed, nor other evidence of neoplastic disease.

Two fine needle aspiration biopsies (FNAs) were performed. The specimens were cellular and consisted of groups of epithelial cells in flat sheets and papillary-like clusters. The cells showed enlarged nuclei with nuclear grooves and a few intranuclear inclusions. Immunohistochemistry on the cell block was positive for TTF-1 (thyroid transcription factor 1), consistent with cells of thyroid origin.

**Fig. 3:**
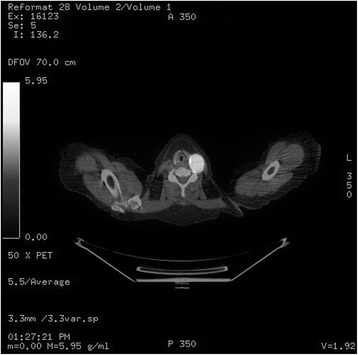
Combined CT/PET capture of fluorodeoxyglucose (FDG) uptake in left lateral neck mass

A positron emission tomography (PET) study was performed to rule out distant metastatic disease. Apart from the known mass in the left neck, the PET study showed no other Fluorodeoxyglucose (FDG) avid lesions (Fig. 3). Given these diagnostic findings, the patient was scheduled for a left lateral neck dissection after obtaining informed consent. The patient subsequently underwent an uncomplicated left level II-III neck dissection under general anesthesia. A timeline of events is presented in Table 1.

**Table 1:** Timeline

1972	Total thyroidectomy, Radioactive iodine ablation
03/2015	Presents to clinic with 2 month history of neck mass
03/2015	Ultrasound identifying solid mass, appropriately unable to detect thyroid
04/2015	CT head, neck, and thorax identifying left-sided level III mass
04/2015	FNA performed on level III mass with immunohistochemical features consistent with metastatic papillary thyroid cancer
05/2015	PET/CT Scan performed with FDG (orbits to the mid-thighs) Local regional recurrence of papillary thyroid cancer
07/2015	Left selective neck dissection level III

Two of the 10 nodes sent for surgical pathology in the lymphadenectomy specimen returned positive for metastatic papillary thyroid carcinoma of tall cell variant. The larger of the 2 nodes measured 3.5 cm with focal extranodal extension.

The patient recovered well from the operation and was seen for follow-up three weeks following the left neck dissection. Given the findings from surgical pathology, it was decided that the patient would benefit from radioactive iodine ablation and the patient was referred for adjuvant therapy.

## Discussion and conclusions

This patient had a distant history of well-differentiated papillary thyroid cancer, which was diagnosed and treated at the age of 28. Her treatment consisted of a total thyroidectomy with post-operative radioactive iodine ablation therapy. Several scores have been developed to stratify patients’ risk of recurrence. These scores broadly take into account patient factors, tumour characteristics, and the selected initial therapy. This patient would have been considered low risk for recurrence by multiple scoring systems: the European Organization for Research and Treatment of Cancer (EORTC) described by Bryar et al. [11]; The Age, Grade, Extent and Size (AGES) score developed by Hay [12]; Age, Metastasis, Extent and Size (AMES) score as described by Cady and Rossi [8]; and the distant Metastasis, patient Age, Completeness of resection, local Invasion, and tumour Size (MACIS) score [13]. More recent findings of associations with mutations in the BRAF^V600E^ gene may in the future serve as a better prognosticator of distant PTC recurrence [14–16].

A handful of studies spanning several decades following postoperative thyroid cancer patients demonstrate that the majority of recurrences occur within the first decade, and that there is a precipitous drop in the incremental recurrence rate in each subsequent decade. These studies and a few case reports, however, do highlight that recurrence as late as 39 years can occur. The case presented in this report details a recurrence 43 years following treatment.

The extent of postoperative follow-up for patients having received thyroidectomy is informed by the relatively low rates of recurrence beyond the initial decade and the surveillance cost of decades-long follow-up. The need to extend surveillance to these later years is not addressed in this report and the benefit of doing so is the subject of further study. However, the findings from this case serve to inform the head and neck surgeon that recurrence has now been demonstrated to occur into the 5th decade following therapy. The authors recommend that PTC recurrence be considered high on the differential diagnosis for a new neck mass, regardless of the elapsed time following initial treatment, until proven otherwise.

## Abbreviations

AGES: Age, Grade, Extent and Size
AMES: Age, Metastasis, Extent and Size
CT: Computed Tomography
EORTC: European Organization for Research and Treatment of Cancer
FDG: Fluorodeoxyglucose
FNA: Fine Needle Aspiration
MACIS: Metastasis, patient Age, Completeness of resection, local Invasion, and tumour Size
PET: Positron Emission Tomography
PTC: Papillary Thyroid Carcinoma
TTF-1: Thyroid Transcription Factor 1
